# Prediction of plasma volume and total hemoglobin mass with machine learning

**DOI:** 10.14814/phy2.15834

**Published:** 2023-10-12

**Authors:** B. Moreillon, B. Krumm, J. J. Saugy, M. Saugy, F. Botrè, J. M. Vesin, R. Faiss

**Affiliations:** ^1^ Research and Expertise in anti‐Doping Sciences (REDs), Institute of Sport Sciences University of Lausanne Lausanne Switzerland; ^2^ Union Cycliste Internationale World Cycling Centre Aigle Switzerland; ^3^ Laboratorio Antidoping Federazione Medico Sportiva Italiana Rome Italy; ^4^ Signal Processing Laboratory 2 Swiss Federal Institute of Technology Lausanne Switzerland

**Keywords:** blood, machine learning, plasma volume, prediction, total hemoglobin mass

## Abstract

Hemoglobin concentration ([Hb]) is used for the clinical diagnosis of anemia, and in sports as a marker of blood doping. [Hb] is however subject to significant variations mainly due to shifts in plasma volume (PV). This study proposes a newly developed model able to accurately predict total hemoglobin mass (Hbmass) and PV from a single complete blood count (CBC) and anthropometric variables in healthy subject. Seven hundred and sixty‐nine CBC coupled to measures of Hbmass and PV using a CO‐rebreathing method were used with a machine learning tool to calculate an estimation model. The predictive model resulted in a root mean square error of 33.2 g and 35.6 g for Hbmass, and 179 mL and 244 mL for PV, in women and men, respectively. Measured and predicted data were significantly correlated (*p* < 0.001) with a coefficient of determination (*R* ^2^) ranging from 0.76 to 0.90 for Hbmass and PV, in both women and men. The Bland–Altman bias was on average 0.23 for Hbmass and 4.15 for PV. We herewith present a model with a robust prediction potential for Hbmass and PV. Such model would be relevant in providing complementary data in contexts such as the epidemiology of anemia or the individual monitoring of [Hb] in anti‐doping.

## 1 INTRODUCTION

Hemoglobin (Hb) is an essential iron‐containing protein present in red blood cells (RBC). Its levels in the body are usually reported in concentration (g/L or g/dL) as RBC are suspended in plasma and are mostly measured as part of a complete blood count (CBC). CBC is a rapid and relatively inexpensive method that offers important information regarding the diagnosis of different pathologies or conditions. Notably CBC, and hemoglobin concentration ([Hb]), can be used to detect different “blood doping” strategies (Breenfeldt Andersen et al., 2022; Jelkmann & Lundby, 2011), to identify individuals at risk of stroke in vulnerable populations (Chang et al., 2020; Panwar et al., 2016), to help patients hospitalization decision (Shimoni et al., 2019) or to regulate patient blood management (PBM) (Desai et al., 2018; Mueller et al., 2019; Murphy & Goodnough, 2015). CBC is also used to diagnose hemoglobin disorders such as anemia. Anemia is a condition defined by [Hb] under certain reference ranges and can be due to a variety of causes such as nutritional deficiencies, inflammations, infections, and bleeding or genetic conditions. The World Health Organization (WHO) established thresholds from 11 g/dL to 13 g/dL (depending on age and sex) for its diagnosis (WHO, 2011) and its prevalence rate can exceed 70% in children under 5 years in some African and Asian countries (WHO, 2023a). Thus, early diagnosis is important to prevent chronic impairments such as bone disease, liver and spleen enlargement, growth impairment, and brain and cardiac dysfunctions (Allali et al., 2017; Cascio & DeLoughery, 2017; Deivita et al., 2021).

Numerous studies demonstrated the pre‐analytical, analytical, and physiological variability of [Hb] measurement (Berkow, 2013; Karakochuk et al., 2019). [Hb] is highly dependent on plasma volume (PV) which is regulated by the renin–angiotensin‐aldosterone system, and prone to significant fluctuations. Indeed, several studies demonstrated changes in PV in a variety of conditions such as heat exposure, hypoxia, acute and chronic physical activities, or hydration status (Sawka et al., 2000; Young et al., 2019; Zouhal et al., 2021). Thus, the information provided by CBC and [Hb], albeit robust, can be difficult to interpret for experts due to the latter confounding factors (Otto et al., 2017).

While [Hb] is highly dependent on PV and its variability, total hemoglobin mass (Hbmass) is a relatively stable parameter. It represents the absolute mass of circulating hemoglobin in the body and several studies confirmed its stability in different situations, notably during altitude training camps or with exercise protocols (Eastwood et al., 2008; Garvican et al., 2010; Hauser et al., 2017; Nummela et al., 2021; Pottgiesser et al., 2009). In the context of medical diagnosis, Hbmass measurement provides complementary and relevant information to the CBC. Current gold standard methods to measure Hbmass are the infusion of radioactive isotope tracers, such as sodium pertechnetate and sodium radiochromate (Gray & Sterling, 1950; Thomsen et al., 1991), or the use of fluorescent dyes, such as indocyanine green (Jones & Wardrop, 2000), which are expensive and time‐consuming. More recently, the carbon monoxide (CO) rebreathing method has been improved and scientifically approved. Briefly, it consists of rebreathing during a brief period (2–10 min) a small bolus of pure CO, which has a much greater affinity to hemoglobin compared to oxygen, mixed with O_2_ in a closed circuit. By assessing the increase in carboxyhemoglobin after rebreathing, Hbmass and PV can be quantified (Burge & Skinner, 1995; Schmidt & Prommer, 2005; Siebenmann et al., 2017). However, this method is rather time‐consuming and more expensive than CBC. Moreover, this method raises some practical and safety challenges both in medical patients with conditions such as heart failure or coronary artery disease (Ahlgrim et al., 2014; Ahlgrim et al., 2018; Karlsen et al., 2016; Wachsmuth et al., 2019) and needs to be adapted to be routinely applied as part of the anti‐doping tests on athletes.

CBC is thus, a rapid and robust method that gives relevant information for the diagnosis of numerous medical conditions, but which is subject to potential high variability due to PV shifts. Hbmass measurement offers valuable complementary information, but is more challenging, especially from a logistical point of view. Therefore, the aim of this study was to design a computational model using machine learning to estimate Hbmass and PV from the variables included in a simple CBC measurement with the hypothesis that measured and predicted values would be in good agreement.

## 2 MATERIALS AND METHODS

### 2.1 Study subjects

Seven hundred and sixty‐nine data points with concomitant CBC and direct blood volume measurements were collected from different studies in which participants volunteered to take part. Briefly, populations consisted of (i) healthy and physically active men and women (*n* = 44), (ii) breath holders (apneists) (*n* = 15), and (iii) elite athletes (*n* = 31). No particular exclusion criteria were expressed. More anthropometric data are displayed in Table 1. In total, 330 observations in women and 439 observations in men were collected. Procedures and risks were fully explained to the subjects who provided their written consent to participate in the study. All studies were approved by the local ethics committee (Agreement 2018‐01019, CER‐VD, Lausanne, Switzerland) and conducted in accordance with the Declaration of Helsinki.

**TABLE 1:** Mean ± standard deviation (SD) for anthropometric, hematological (hemoglobin concentration ([Hb]), and hematocrit values.

	Control men (*n* = 123)	Control women (*n* = 255)	Apneists men (*n* = 75)	Apneists women (*n* = 25)	Elite men (*n* = 241)	Elite women (*n* = 50)
Age (years)	28.1 ± 6.7	23.1 ± 1.9	37.8 ± 7.7	39.6 ± 8.5	248 ± 4.1	25.6 ± 2.7
Height (cm)	178 ± 4	166 ± 5	182.0 ± 8.0	169.1 ± 3.5	181.0 ± 5.6	169.5 ± 4.2
Mass (kg)	72.2 ± 8.9	61.3 ± 6.1	76.4 ± 9.4	62.8 ± 2.8	73.6 ± 5.6	63.0 ± 5.2
[Hb] (g/dL)	14.8 ± 1.0	12.9 ± 0.9	14.6 ± 0.9	13.1 ± 0.5	14.7 ± 0.9	13.7 ± 0.9
HCT (%)	43.3 ± 2.5	38.2 ± 2.0	41.7 ± 2.1	38.2 ± 1.7	42.9 ± 2.7	41.0 ± 2.6

## 3 BLOOD SAMPLING

Blood sampling for the measurement of the Athlete Biological Passport (ABP) hematological module was conducted by two experienced phlebotomists following the current World Anti‐Doping Agency (WADA) blood analytical requirements for the ABP (WADA, 2021). The participants reported to the lab having avoided any physical exercise in the 2 h preceding the sampling. The subjects then remained seated for 10 min to avoid an acute PV variation (Astolfi et al., 2020). Whole blood was collected either in 2‐mL tubes (BD Vacutainer® tubes (EDTA‐K2 (K2) CE cat no. 368856/ref US 367856, BD Europe) or 2.7‐mL tubes (K2 EDTA 2.7 mL, Sarstedt AG) considered equivalent, which were homogenized at room temperature on a roller for 15–45 min, and subsequently analyzed in duplicate by flow cytometry (Sysmex XN‐1000, Sysmex). Internal quality controls provided by the manufacturer (Sysmex E‐Checks, levels 2 and 3) were run twice before and after each batch of samples. The following variables were measured: white blood cell count (WBC#), RBC, [Hb], hematocrit (HCT), mean corpuscular volume (MCV), mean corpuscular hemoglobin (MCH), mean corpuscular hemoglobin concentration (MCHC), platelets (PLT), red blood cell distribution width (standard distribution) (RDW‐SD), red blood cell distribution width (coefficient of variation) (RDW‐CV), platelet distribution width (PDW), mean platelets volume (MPV), platelet‐large cell ratio (P‐LCR), procalcitonin (PCT), neutrophils count (NEUT#), lymphocytes count (LYMPH#), monocytes count (MONO#), eosinophils count (EO#), basophiles count (BASO#), neutrophils percentage (NEUT%), lymphocytes percentage (LYMPH%), monocytes percentage (MONO%), eosinophils percentage (EO%), basophiles percentage (BASO%), immunoglobulins count (IG#), immunoglobulins percentage (IG%), reticulocytes percentage (RET%), reticulocytes count (RET#), and immature reticulocyte fraction (IRF).

### 3.1 Plasma volume and total hemoglobin mass measurement

A fully automated system was used to determine PV and HBmass using a CO rebreathing procedure (OpCo: Detalo Instruments, Birkerod), which is outlined in detail elsewhere (Siebenmann et al., 2017). Briefly, subjects were asked to breathe a 100% oxygen (O_2_) in a closed circuit before a bolus of 1 mL/kg of 99.997% chemically pure CO (Carbagas) was administered in the circuit and rebreathed for 9 min in supine position with elevated feet to allow for an optimal whole body blood circulation during rebreathing. Venous blood samples of 1.2 mL (S‐Monovette Li‐Heparin, Sarstedt) were taken before and after the rebreathing phase for the determination of carboxyhemoglobin (HbCO%) in triplicate with a separate gasometer (ABL80 Co‐Ox, Radiometer) for HBmass determination using formulas described elsewhere (Siebenmann et al., 2017). The CO remaining in the system was measured with a CO meter (Monoxor Plus, Bacharach) and subtracted from the initial amount introduced to define the exact CO bolus received with a 0.1 mL typical error. The total red blood cell volume (RBCV), PV, and blood volume (BV) were then calculated from HBmass, [Hb], and hematocrit (HCT). In this study, the total error for the determination of HBmass was 1.8%, in line with existing studies (Siebenmann et al., 2017).

### 3.2 MATLAB regression

The modeled data were calculated using MATLAB regression learner app (MATLAB R2022b, The MathWorks, Inc.). Anthropometric data (age [y], height [cm], and weight [kg]) and CBC (listed above) data were compiled and imported into MATLAB to be used as variables to predict PV and Hbmass with actually measured Hbmass and PV for the paired data as targets for the estimates. All data points (*n* = 769) were used in the calculation of the prediction model. The models were evaluated using 10‐fold cross‐validations. Additionally, all variables provided by the CBC were used in the regressions to account for as much individual variation as possible and missing data have been previously excluded. Data was discriminated depending on sex. Four different models were created to predict the following variables: Hbmass women, Hbmass men, PV women, and PV men. All machine learning models available through MATLAB regressions learner app were tested: (a) linear regression models; (b) regression trees; (c) support vector machines; (d) Gaussian process regression models; (e) ensemble of trees; and (f) neural networks (Mathworks, 2023a). In total, 24 different models were tested and their respective performance in predicting Hbmass and PV in women and men are displayed in Table 2. A Gaussian Process Regression (GPR) with a constant basis function, a non‐isotropic rational quadratic kernel function, an automatic observation noise standard deviation Sigma, standardized predictors, and optimized numeric parameters (Mathworks, 2023a; Mathworks, 2023b; Mathworks, 2023c; Rasmussen & Williams, 2005) was determined as the best fitting and most consistent model for the four predicted variables with the lowest root mean square error (RMSE) and highest coefficient of determination (*R* ^2^) for all four groups (i.e., Hbmass women, PV women, Hbmass men, and PV men). It was therefore the model used for further analyses.

**TABLE 2:** Root mean square error (RMSE) and coefficient of correlation (*R* ^2^) for all machine learning models tested for the prediction of total hemoglobin mass and plasma volume (PV) in women and men. The lowest RMSE and the highest *R* ^2^ are highlighted in orange.

		Hbmass women		PV women		Hbmass men		PV men	
Model	Function	RMSE (g)	*R* ^2^	RMSE (mL)	*R* ^2^	RMSE (g)	*R* ^2^	RMSE (mL)	*R* ^2^
Linear regression models	Linear	56.3	0.52	262	0.49	77.1	0.54	354	0.57
	Interactions linear	425.2	−26.44	3010	−66.9	921.2	−64.2	4447	−66.28
	Robust linear	57.7	0.49	265	0.47	78.1	0.53	358	0.56
	Stepwise linear	50.1	0.62	238	0.58	67.8	0.65	338	0.61
Regression trees	Fine tree	52.9	0.58	266	0.47	66.1	0.67	407	0.44
	Medium tree	54.4	0.55	271	0.45	66.6	0.66	397	0.47
	Coarse tree	60.8	0.44	301	0.32	77.2	0.55	410	0.43
Support vector machines	Linear	57.6	0.5	276	0.43	81.9	0.49	364	0.55
	Quadratic	41.6	0.74	209	0.67	61.7	0.71	301	0.69
	Cubic	44.6	0.7	236	0.58	76.7	0.55	363	0.55
	Fine Gaussian	78.1	0.07	348	0.09	107.5	0.11	520	0.08
	Medium Gaussian	41.7	0.74	197	0.71	55.6	0.76	287	0.72
	Coarse Gaussian	60.0	0.45	300	0.33	81.6	0.49	379	0.51
Gaussian process regression models	**Rational quadratic**	**33.2**	**0.83**	**179**	**0.76**	**35.6**	**0.9**	**244**	**0.8**
	Squared exponential	37.9	0.78	190	0.73	52.9	0.78	280	0.73
	Matern 5/2	37.3	0.79	189	0.73	52.5	0.79	276	0.74
	Exponential	37.9	0.78	192	0.72	52.4	0.79	272	0.75
Ensembles of trees	Boosted trees	45.6	0.68	232	0.6	67.5	0.65	346	0.59
	Bagged trees	42.3	0.73	221	0.63	54.4	0.77	310	0.67
Neural networks	Narrow	71.7	0.22	331	0.18	99.8	0.24	458	0.29
	Medium	95.0	−0.37	453	−0.54	149.1	−0.7	592	−0.18
	Wide	91.7	−0.28	506	−0.92	154.6	−0.82	754	−0.92
	Bilayered	69.2	0.27	342	0.12	93.6	0.33	441	0.34
	Trilayered	75.2	0.14	347	0.1	90.7	0.37	469	0.26

## 4 STATISTICAL ANALYSES

**FIGURE 1:**
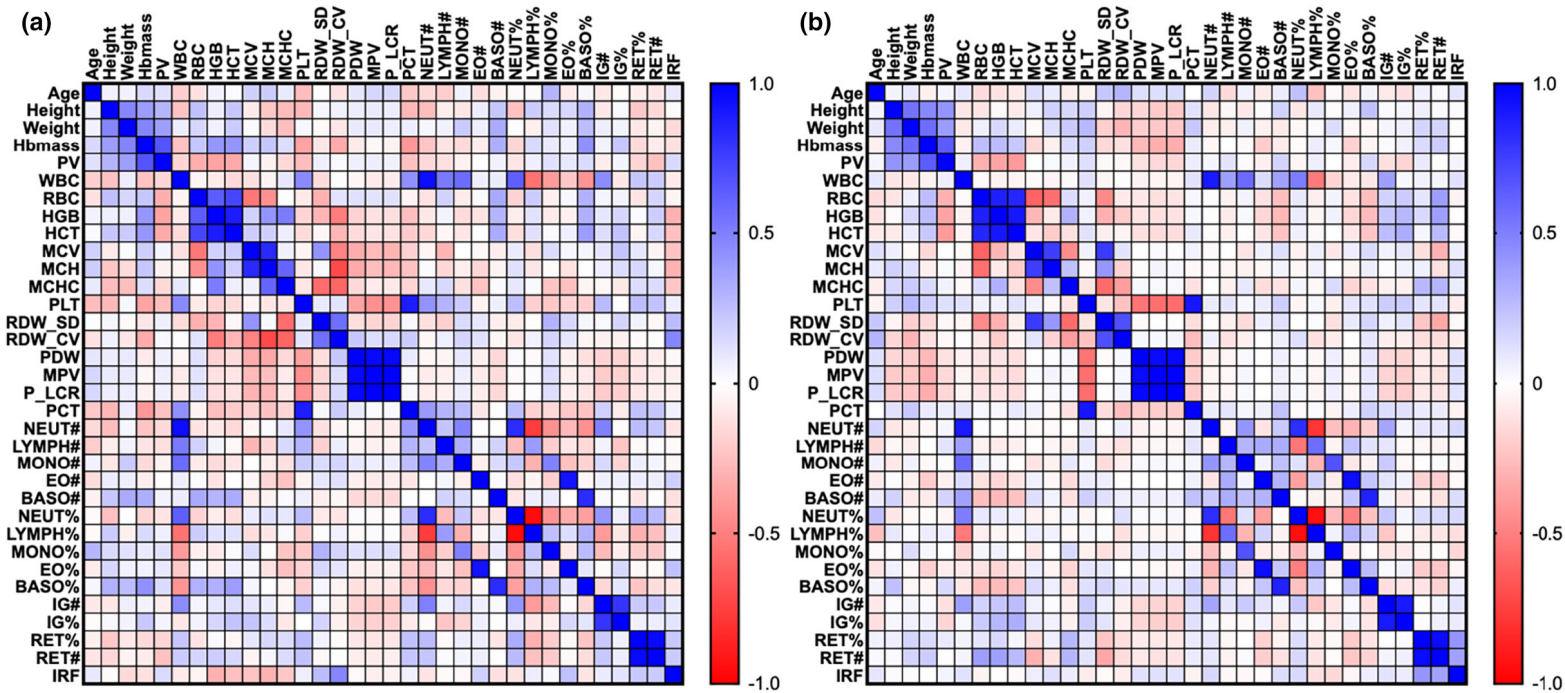
Heatmap of Pearson correlation matrix for (a) men variables and (b) women variables.

Data are presented as means ± standard deviations (SD). Dependency between variables was assessed through a Pearson correlation matrix (Figure 1). To assess agreement between measured and predicted values of Hbmass and PV, RMSE, mean square error (MSE), and mean absolute error (MAE) were calculated. Additionally, Pearson's correlations and Bland–Altman analyses were performed. All statistical analyses were performed with dedicated software (MATLAB R2022b, MathWorks, Inc.; Prism, Version 8.4.2, GraphPad Software).

## 5 RESULTS

**FIGURE 2:**
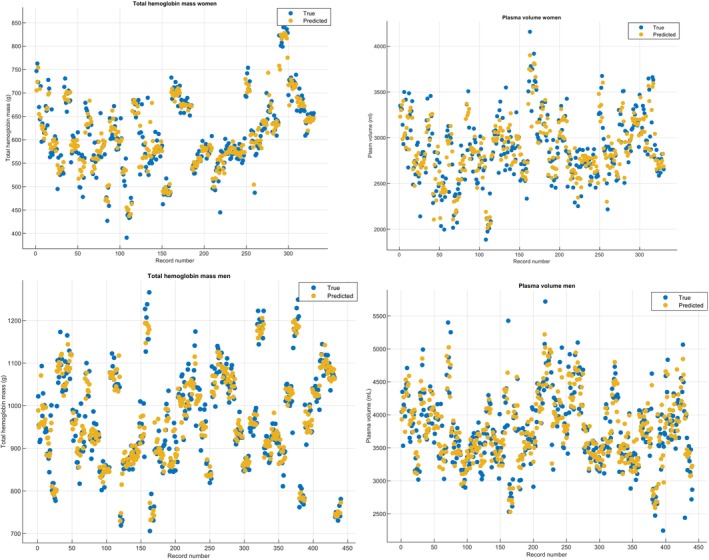
Graphical representations of true (black) versus predicted (gray) total hemoglobin mass (Hbmass) and plasma volume (PV) for women (a, b, respectively) and men (c, d, respectively).

**FIGURE 3:**
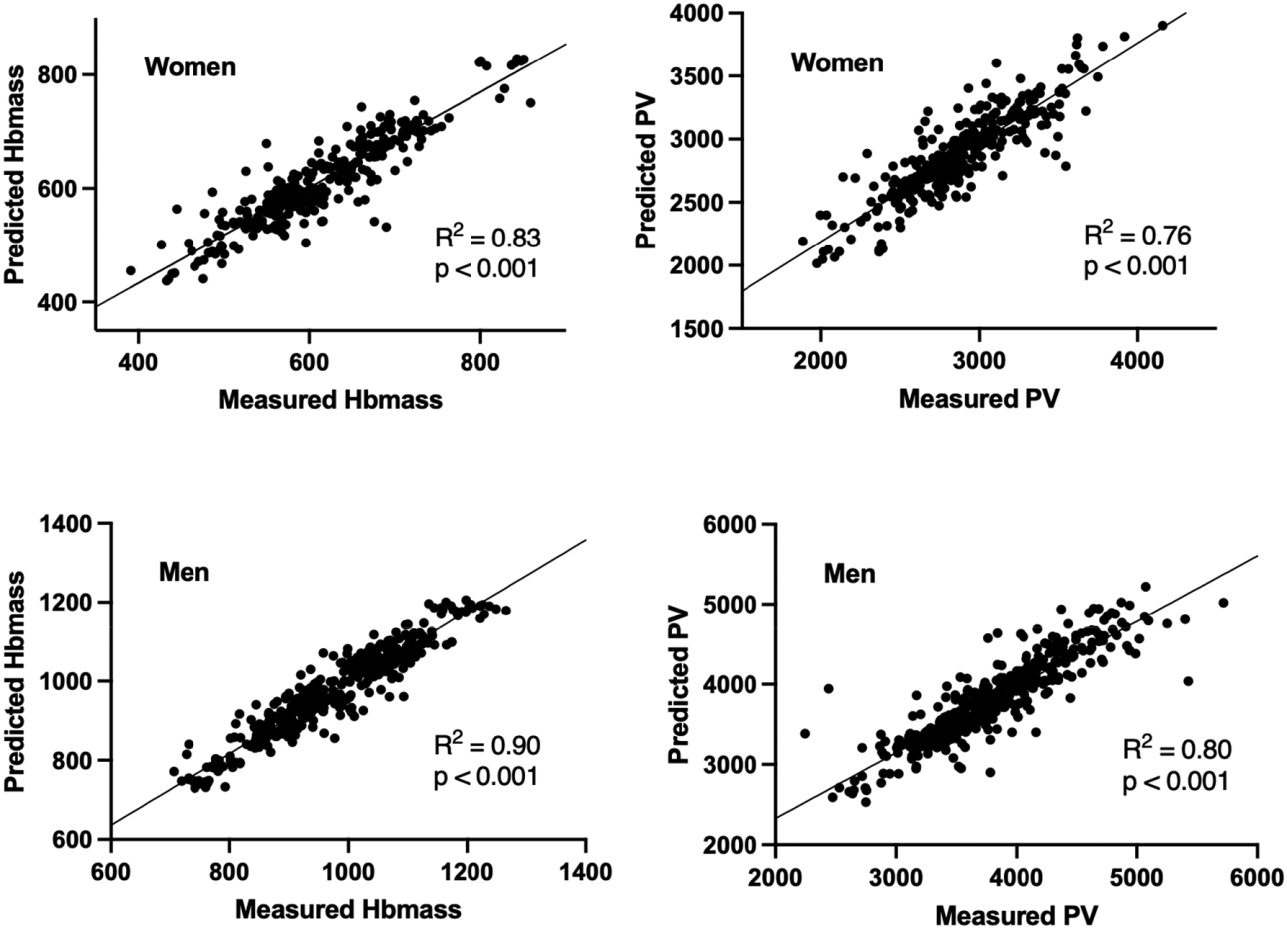
Correlations between measured and predicted total hemoglobin (Hbmass) (left) and plasma volume (PV) (right) for women and men, respectively.

**FIGURE 4:**
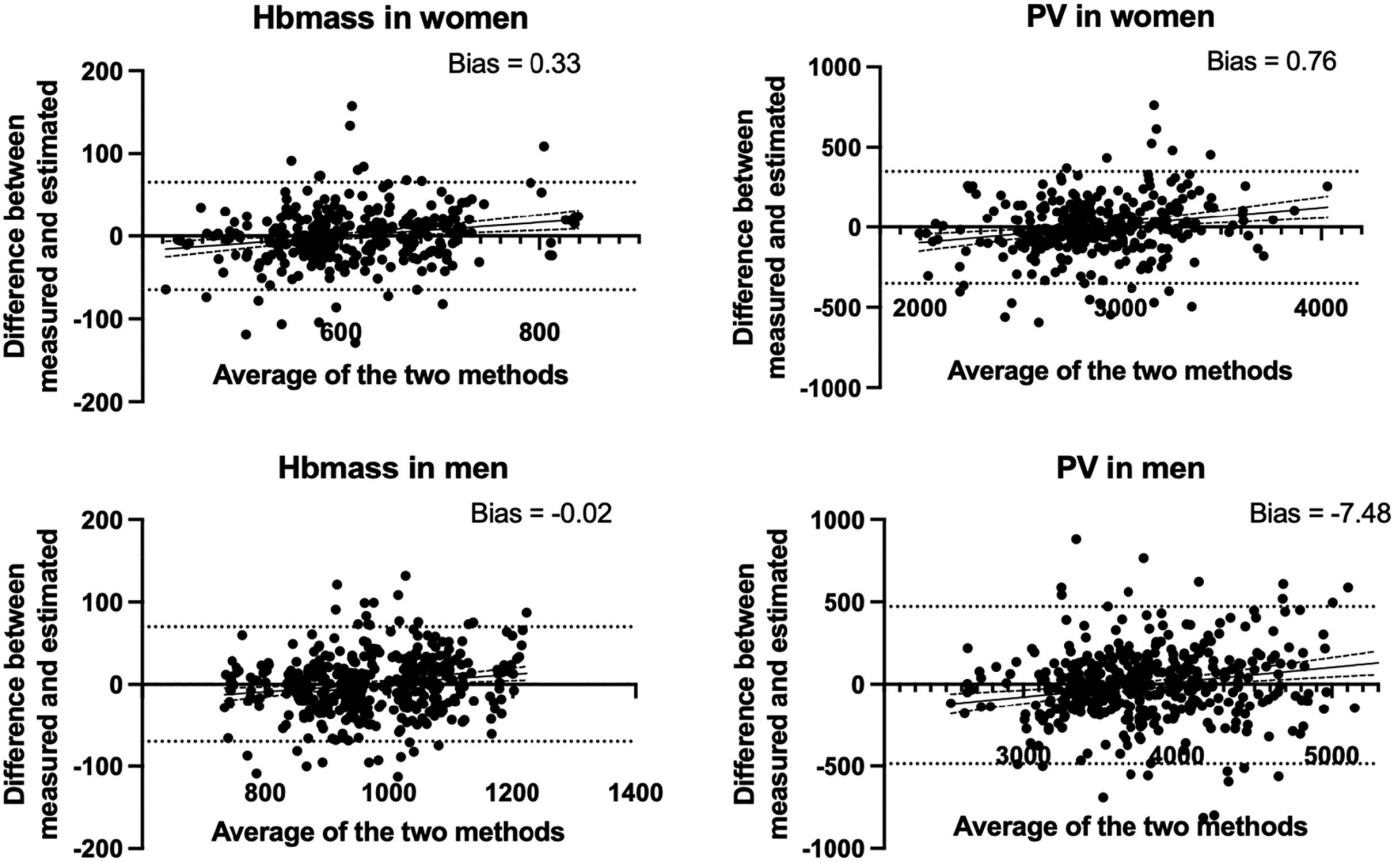
Bland–Altman plots comparing measured and predicted total hemoglobin (Hbmass) and plasma volume (PV) for women and men, respectively. The difference is represented as a function of the average values with 95% limits of agreement (dotted lines), computed as the mean difference (bias) ±1.96 times its SD; and regression fit of the differences on the means (as a solid black line with 95% confidence intervals).

Mean measured Hbmass was 610 ± 82 g for women and 968 ± 114 g for men. Mean measured PV was 2987 ± 1461 mL for women and 3774 ± 543 mL for men (Figure 2). In comparison with estimated values RMSE were of 3.7%, 5.4%, 6.5%, and 6.0%, for Hbmass and PV, in men and women, respectively (Table 3). MAE, which tends to be less sensitive to outliers, was lower in average, albeit non‐significant, than RMSE for every parameter (Table 2). Measured and predicted values for Hbmass and PV were significantly correlated (*p* < 0.001), and *R* ^2^ were 0.83, 0.90, 0.76, and 0.80 for Hbmass women, Hbmass men, PV women, and PV men, respectively (Figure 3). Finally, Bland–Altman's analyses showed that both methods were in good agreement with biases of 0.33, −0.02, −0.81, and −7.48 for Hbmass women, Hbmass men, PV women, and PV men, respectively (Figure 4).

**TABLE 3:** Root mean square error (RMSE), mean square error (MSE), and mean absolute error (MAE) for total hemoglobin mass (Hbmass, g) and plasma volume (PV, mL) predictions in men and women obtained with the rational quadratic Gaussian process regression model.

	PV	Hbmass
Men		
RMSE	244	35.6
MSE	59,667	1270
MAE	168	27.1
Women		
RMSE	179	33.2
MSE	31,882	1104
MAE	130	23.8

Red cell variables (RBC, HGB, and HCT), platelets size variables (PDW, MPV, and P‐LCR), and reticulocytes variables (RET%, RET#, and IRF) are, respectively, strongly (*R* ^2^ = [0.7:1]) and significantly (*p* < 0.001) correlated together in both women and men. Additionally, height and weight are moderately (*R* ^2^ = [0.3:0.6]) correlated with both Hbmass and PV in both women and men.

## 6 DISCUSSION

Hbmass and PV were accurately predicted for both women and men based on anthropometric and CBC analyses using a model calculated with MATLAB's regression learner application. Such a model can be of significant interest in a wide range of applications. Given the low relative RMSE for women and men in predicting Hbmass and PV, this model adds valuable information to the simple CBC in many contexts. The low systematic biases between measured and estimated values support a very good agreement between both methods to determine Hbmass and PV.

It is interesting to note that the Gaussian process regressions models perform the best among all models available in MATLAB regression learner app. When used with a non‐isotropic rational quadratic kernel function, it yields the lowest RMSE and the highest *R* ^2^ for all four groups among all the models available in the MATLAB regression learner app.

Correlations between height and weight are only moderate which is lower than expected from the literature (Falz et al., 2019). While they still remain the two variables explaining the most variance, it highlights the fact that a significant amount of individual variance resides in the other variables provided by the CBC.

Because PV changes are not considered in the [Hb] thresholds established by the WHO to diagnose anemia, this diagnostic model presents substantial limitations (Berkow, 2013; Karakochuk et al., 2019). Numerous studies have soundly measured reference hematological values, including [Hb], in populations with high anemia prevalence (Béavogui et al., 2020; Buchanan et al., 2010; Dapper et al., 2009; Fondoh et al., 2020; Humberg et al., 2011; Kone et al., 2017; Schmidt et al., 2018; Umar et al., 2015;WHO, 2023a; WHO, 2023b). The data provided by these studies, despite being crucial regarding anemia epidemiology, can possibly lead to over‐ or underestimation of total prevalence due to PV fluctuations. For instance, individuals originating from regions with large climatic seasonal differences or very warm temperatures do present different regulatory adaptations regarding body fluids with increased water retention (Tochihara et al., 2022). This could possibly lead to greater hemodilution and to overestimation of anemia prevalence in the latter populations which constitute the majority of the countries with severely high prevalence according to the WHO (WHO, 2023a; WHO, 2023b). Additionally, current WHO guidelines suggest adding from 0.2 g/dL for people living at 1000 m to 4.5 g/dL for people living at or over 4500 m to the population thresholds to account for the increase in RBC synthesis due to the hypoxic environment (Cohen & Haas, 1999; WHO, 2011). However, this practice does not relate accurately to the specific population patterns of adaptations to high altitude and could lead to misdiagnosis (Choque‐Quispe et al., 2020; Gonzales et al., 2018; Sarna et al., 2018; Sarna et al., 2020). It has been shown that Andeans population living at extremely high altitude such as the Aymaras present exceptionally high [Hb] (~19.1 g/dL), whereas Tibetans and Ethiopians living at similar altitudes have far lower mean values (~15.8 g/dL and 15.6 g/dL, respectively) (Beall, 2006; Beall et al., 1998). This clearly indicates divergent functional adaptations to similar highly hypoxic environments in those populations (Beall, 2007). Providing accurate Hbmass and PV estimation would thus, support anemia diagnosis in high altitude populations and help discern between hemoglobin synthesis and volumetric adaptations (Stembridge et al., 2019). Ultimately, [Hb] thresholds or Hbmass and PV estimations as presented here are only mere screening tools and final diagnosis of anemia require more advanced testing to accurately diagnose the disease and its cause.

In addition, this model could prove useful in an anti‐doping scenario, especially in the framework of the ABP. The hematological module of the ABP is a tool which longitudinally monitors a number of individual hematological parameters in order to detect a normal variations potentially indicative of doping (Saugy & Leuenberger, 2020; Sottas et al., 2010). [Hb] being a primary biomarker, the ABP hematological module is subject to multiple confounding factors impacting PV, and in turn [Hb], such as exercise, heat or altitude exposure (Krumm & Faiss, 2021). Indeed, it has been demonstrated that both acute and multiday exercise can significantly impact PV (Astolfi et al., 2021; Gore et al., 2013). Additionally, nowadays, heat and hypoxic training are widely used to elicit ergogenic hematological adaptations, but prompting at the same time significant shifts in body fluids (Baranauskas et al., 2021). For instance, hypoxic training improves Hbmass due to an increase in erythropoietin (EPO) production (Baranauskas et al., 2021); but altitude training also induces severe hemoconcentration, noticeably, Young and colleagues demonstrated a decrease in PV larger than 15% after 19 days of continuous exposure (Young et al., 2019). On the opposite, heat training induces large increase in PV as a response to the increase of body temperature and can arguably increase Hbmass as well (Baranauskas et al., 2021). In this context, physiological variations can be hard to discern from actual doping when only longitudinally monitoring [Hb].

To assess longitudinal PV variations, Lobigs and colleagues developed and validated a predictive model based on both hematological and serum biomarkers able to explain up to 67% of the variance (Garvican‐Lewis et al., 2020; Lobigs et al., 2017; Lobigs, Garvican‐Lewis, et al., 2018; Lobigs, Sottas, et al., 2018). This model proved useful to reduce false‐positive rate by adjusting the ABP limits of detection in several studies (Garvican‐Lewis et al., 2020; Lobigs, Garvican‐Lewis, et al., 2018; Moreillon et al., 2022). Despite its interest and potential utility for anti‐doping purposes, this model is hardly applicable on a large scale due to the costs and technical challenges of serum sampling (Miller et al., 2019). Conversely, the model presented here explains respectively 74% and 75% of PV variance in women and men, and do not require any supplementary analysis or additional costs. It could therefore easily be implemented in anti‐doping policies worldwide and provide valuable information to the expert reviewing the hematological module of the ABP.

However, the model introduced here presents some limitations. The fact that the prediction performance metrics are assessed on the same data used to fit the model can potentially lead to overfitting which can only be partially limited by the 10‐fold cross‐validation process. Second it also presents limitations regarding its use in a medical context. Indeed, it was trained on data obtained from healthy and physically active subjects, and it questions its application in unhealthy populations. There is thus, a need for data acquired in more comprehensive cohorts to validate the model for medical purposes. The machine‐learning paradigm also allows the model to be trained with additional data from diverse cohorts for a more precise and fine‐tuned real‐life application.

In conclusion, estimation of Hbmass and PV based on CBC and anthropologic analyses offers highly relevant complementary data about hematological parameters. The model proposed can prove to be valuable in a wide variety of contexts, from the diagnosis of widespread diseases to anti‐doping with the convenient advantages of being inexpensive, timesaving, and easy to implement.

## AUTHOR CONTRIBUTIONS

Moreillon B., Vesin J.M., Krumm B., and Faiss R. designed the study. Moreillon B., Krumm B., Saugy J.J., Saugy M., and Faiss R. contributed to data collection. Moreillon B. and Vesin J.M. performed the regression analyses. All authors reviewed the results. Moreillon B. drafted the first version of the manuscript, and all authors revised it critically. All authors read and approved the final version of the manuscript.

## ETHICS STATEMENT

Procedures and risks were fully explained to the subjects who provided their written consent to participate in the study. All studies were approved by the local ethics committee (Agreement 2018‐01019, CER‐VD, Lausanne, Switzerland) and conducted in accordance with the Declaration of Helsinki.

## FUNDING INFORMATION

This research received no specific grant from any funding agency.

## CONFLICT OF INTEREST STATEMENT

The authors have no conflicts of interest to declare.

## Data Availability

The data underlying this article will be shared on reasonable request to the corresponding author. Predictive models can be found here: https://github.com/Bmoreillon/Hbmass‐and‐PV‐prediction‐model/blob/main/README.md
